# Topological defects law for migrating banded vegetation patterns in arid climates

**DOI:** 10.1126/sciadv.adf6620

**Published:** 2023-08-04

**Authors:** D. Pinto-Ramos, M. G. Clerc, M. Tlidi

**Affiliations:** ^1^ Departamento de Física and Millennium Institute for Research in Optics, FCFM, Universidad de Chile, Casilla 487-3, Santiago, Chile.; ^2^ Faculté des Sciences, Université Libre de Bruxelles, CP 231, Campus Plaine, B-1050 Bruxelles, Belgium.

## Abstract

Self-organization and pattern formation are ubiquitous processes in nature. We study the properties of migrating banded vegetation patterns in arid landscapes, usually presenting dislocation topological defects. Vegetation patterns with dislocations are investigated in three different ecosystems. We show through remote sensing data analysis and theoretical modeling that the number of dislocations *N*(*x*) decreases in space according to the law *N* ∼ log(*x*/*B*)/*x*, where *x* is the coordinate in the opposite direction to the water flow and *B* is a suitable constant. A sloped topography explains the origin of banded vegetation patterns with permanent dislocations. Theoretically, we considered well-established approaches to describe vegetation patterns. All the models support the law. This contrasts with the common belief that the dynamics of dislocations are transient. In addition, regimes with a constant distribution of defects in space are predicted. We analyze the different regimes depending on the aridity level and water flow speed. The reported decay law of defects can warn of imminent ecosystem collapse.

## Teaser

Distribution of defects in vegetation patterns could determine the ecosystem adaptation or collapse against environmental changes.

## INTRODUCTION

Self-organization phenomena leading to spatially periodic patterns are observed in complex or nonlinear systems (*1*–*5*). Vegetation population dynamics provide puzzling and notable examples of spatially periodic structures, generically called vegetation patterns, formed by large-scale botanical organizations controlled by a nonequilibrium symmetry-breaking instability (*6*–*13*). The banded patterns, often called tiger bush (*14*), consist of dense vegetation bands alternating with sparsely covered or even bare soil, their wavelength ranges from decimeters to hundreds of meters. Banded vegetation patterns have probably been first reported by Macfadyen in the earlier fiftieth (*15*, *16*). The spontaneous symmetry-breaking instability causes their formation even when the topography is flat (*6*). The presence of the slope causes the migrating banded patterns (*6*, *8*, *12*). They grow by a few decimeters each year in the opposite direction of the water flow (*8*, *12*). Besides, a bibliography of empirical and scientific studies devoted to the origin of their formation and maintenance can be found in (*6*, *12*, *17*–*20*).

**Fig. 1.**
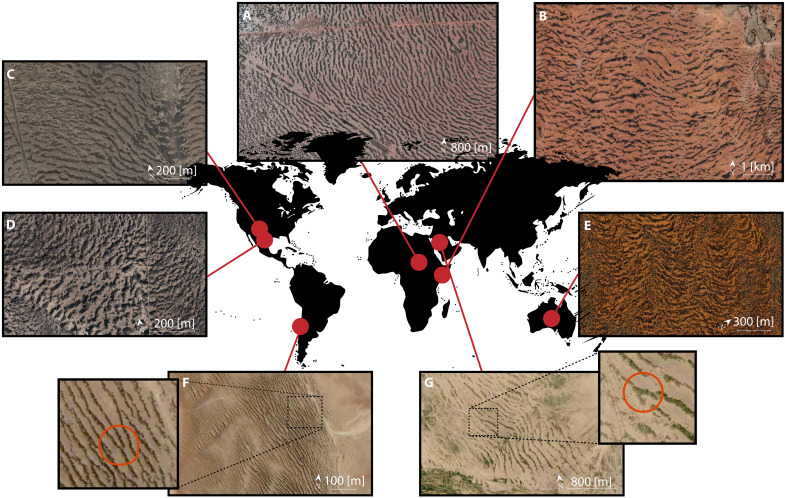
Migrating banded vegetation patterns with dislocations observed in arid and semi-arid ecosystems on different continents. (**A**) Sudan 11° 9′ N, 28° 16.5′ E. (**B**) Somalia 8° 6.9′ N, 47° 26.5′ E. (**C**) United States 31° 2.5′ N, 103° 5.5′ W. (**D**) Mexico 28° 8.5′ N, 104° 28′ W. (**E**) Australia 23° 23′ S, 133° 23.2′ E. (**F**) Chile 20° 29.5′ S, 70° 3.5′ W. (**G**) Saudi Arabia 24° 19.8′ N, 42° 55.2′ E. Insets show dislocations indicated with red rings.

Most of the banded vegetation patterns observed in nature are disordered and present topological defects such as dislocations, as can be seen in Fig. 1. Dislocations in the banded vegetation patterns are indicated by red rings in the aerial photographs of Fig. 1. When two stripes join and transform into a single one, they form a defect called dislocation. Observations across large areas of numerous arid and semi-arid regions of Africa, Australia, America, and the Middle East show that topological defects are abundant. Banded vegetation is a well-documented issue that has been abundantly discussed and is by now fairly well understood. So far, however, the law governing the formation of such defects has neither been experimentally determined nor theoretically predicted.

Here, we establish a law governing the organization of dislocations. By analyzing satellite images taken from vast territories of the African and American continent, we show that the number of dislocations obeys the formula *N* ∼ log(*x*/*B*)/*x*, where *x* is the coordinate in the opposite direction of water flow and *B* is a suitable constant. Theoretically, we have considered three different ecological approaches describing the dynamics of topological defects. All these models quantitatively support this deterministic law. Furthermore, these ecological models predict an additional dynamical regime where the number of dislocations remains constant. In addition to the slope, which is the source of dislocation propagation, we show that boundary conditions play an essential role in their permanent creation; defect generation from boundaries is a documented phenomenon in nonlinear physics that appears in several situations, the most common being the dynamics of viscous flows (*21*). Therefore, with a source of dislocations through the boundaries, the dynamics of these topological defects can be permanent rather than transient. This fact strongly contrasts with previous work where dislocation formation is considered a transient dynamic due to their mutual annihilation interaction, leading at long times to a perfectly ordered banded pattern free of defects (*8*). The permanent dynamics of defects is the process of pairs of dislocations being created at the boundary with opposite topological charges, and then they move with the pattern migration velocity (toward *x*) at the same time they interact, approaching each other until annihilation; the process is repeated in time in an unpredictable way. This complex permanent dynamic leaves an imprint in the dislocation number as a function of the *x* direction. We demonstrate how a decaying number of dislocations in space may be used as an early indicator of an ecosystem’s potential collapse under harsh environmental conditions. We conclude by showing how the measure of the dislocation distribution in space can be used as a noninvasive tool for diagnosing ecosystem health. The ecosystem transition to bare soil is a much-studied issue in which spatial vegetation models play a crucial role (*22*–*24*). Our theory complements the understanding of ecosystem adaptability and resilience until now, as we consider the role of sloped topography and boundary conditions in the dynamics. The predicted law is supported by field observations and can be crucial for identifying and comprehending the different spatiotemporal behaviors seen in complex systems other than ecological ones.

## RESULTS

### Remote sensing data analysis and the dislocation distribution decay law

**Fig. 2.**
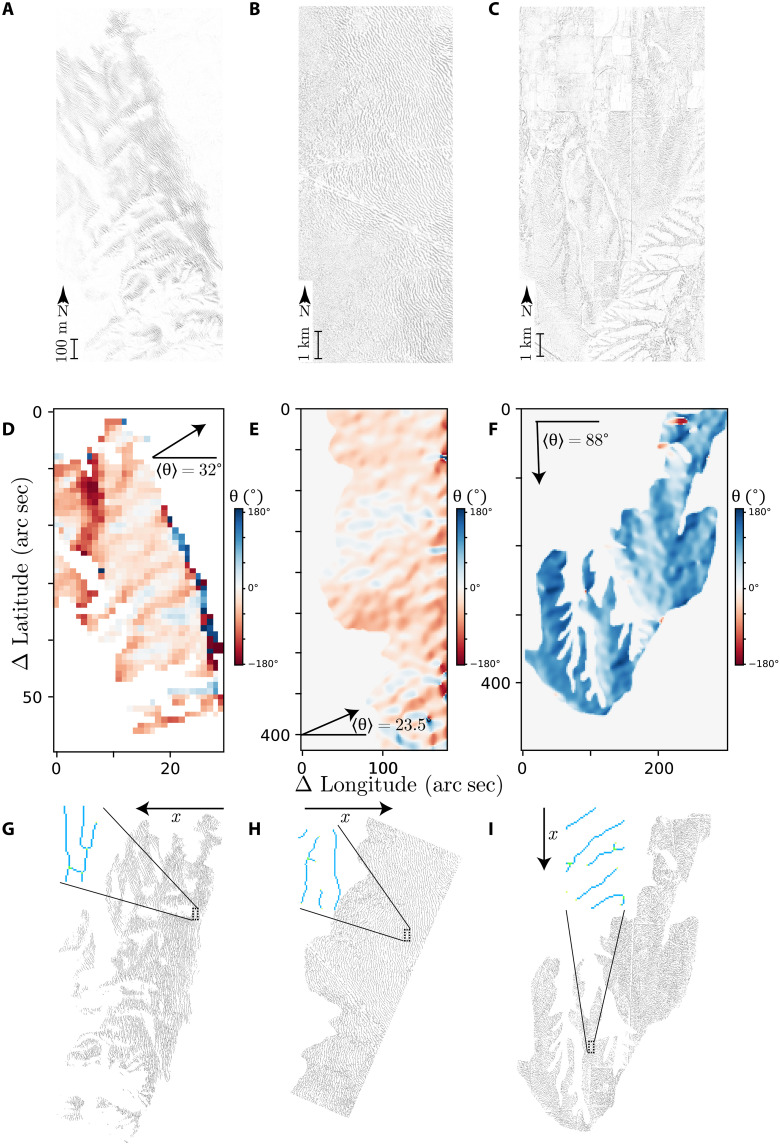
Remote sensing analysis: Determination of the *x* direction and defect recognition with remote sensing data. (**A** to **C**) show the vegetation patterns in Chile 20 ^β^ 29.5′ S, 70° 3.5′ W, Sudan 11° 9′ N, 28° 16.5′ E, and the United States 31° 2.5′ N, 103° 5.5′ W, respectively. (**D** to **F**) exhibit the direction of the steepest variation in the altitude over the region of interest. (**G** to **I**) illustrate the pattern’s skeletons, and insets show the patterns dislocations highlighted.

To establish through field observation that the number of dislocations in the banded vegetation follows a logarithmic law, we perform an image analysis. Three regions of the world are considered: Chile, Sudan, and the United States. To do that, we use high-resolution satellite images obtained from the Google Earth software (https://earth.google.com/web), together with the elevation database SRTM (Shuttle Radar Topography Mission) with 1–arc sec resolution (*25*). First, we select and create an adequate mask of the region where banded vegetation patterns are settled on sloppy landscape as shown in the satellite images of Fig. 2 (A to C). Second, we extract the mean orientation of the elevation gradient 〈θ〉 over the selected region as illustrated by Fig. 2 (D to F). We assume that the mean orientation of the elevation gradient is parallel to *x*. In the case of the banded vegetation pattern in hyper-arid landscapes of Chile, the *x* variable decreases with height, as water comes from the East-to-West traveling fog (*26*, *27*). This means that the water bubbles move uphill, and therefore, the vegetation pattern migrates downhill. However, in arid landscapes of North America and Sudan, water is supplied by rainfall, and the *x* variable grows with height.

Once the *x* direction is defined, dislocation positions are marked. For the satellite images, because of the intrinsic fluctuations, the high anharmonicity, and the high variations in the wavelength in the banded vegetation, the dislocations could not be recognized with standard methods. To detect dislocations, we construct a skeleton of the banded vegetation pattern using the software for scientific image analysis Fiji (*28*) (see Materials and Methods section). This method allows us to identify the branch split points and the branch ends as points representing dislocations of the local pattern. The results are summarized in Fig. 2 (G to I).

**Fig. 3.**
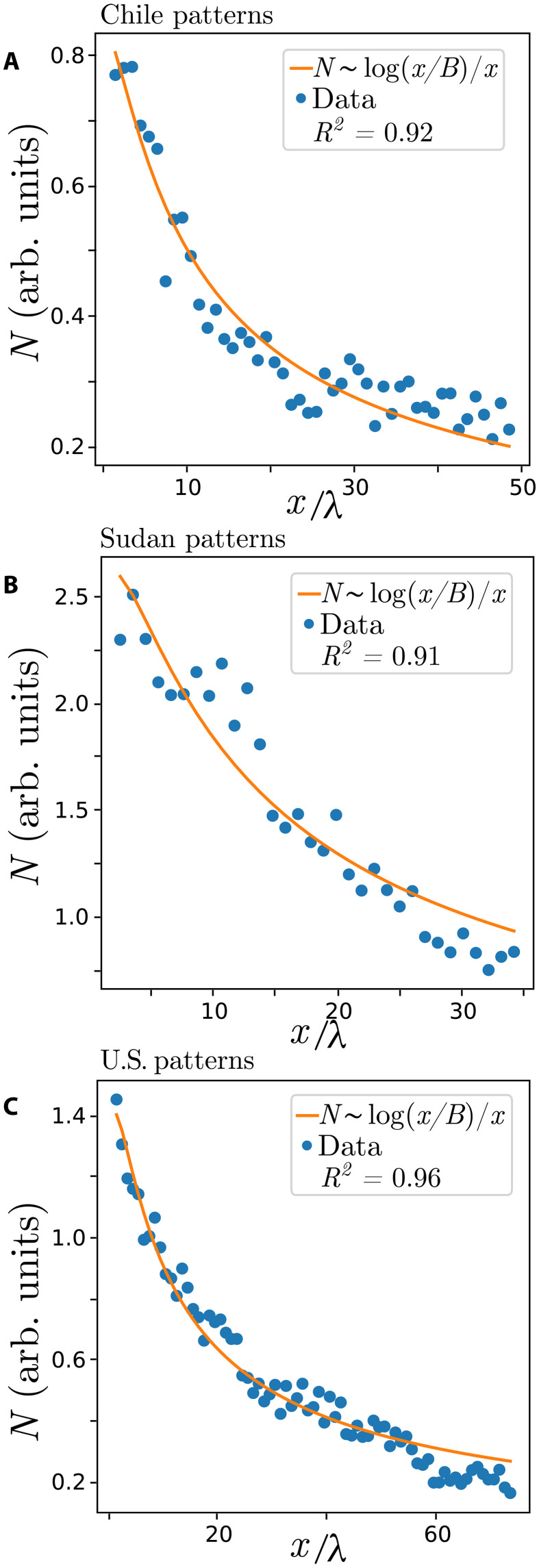
Dislocation number decay law obtained from remote sensing analysis in Chile, Sudan, and U.S. landscapes. Circles account for observed data, and the orange curves represent the fits. (**A** to **C**) correspond to a *N*(*x*) ∼ log(*x*/*B*)/*x* fit for patterns in Chile, Sudan, and the United States, respectively. Fit parameters in λ units are (A) *x*_0_ = −2.9, *B* = 1.2, and *A* = 2.7; (B) *x*_0_ = −2, *B* = 1.5, and *A* = 10.6; (C) *x*_0_ = −2.4, *B* = 1.3, and *A* = 5. *R*^2^ is the coefficient of determination of the fits.

Last, we select an area within the banded vegetation pattern in the plane (*x*, *y*), and we define the dislocation number *N*(*x*, *y*) over tiles of one wavelength side. Then, we average along the *y* direction. The obtained dislocation number *N*(*x*) is plotted as a function of *x*/λ where λ is the wavelength of the banded vegetation pattern. Note that *N* is the expected number of dislocations in a λ^2^ surface tile centered on the (*x*, *y*) plane. The results are shown in Fig. 3. In the hyper-arid landscape of Chile and Sudan and the United States arid landscapes, the number of dislocations *N*(*x*) decreases with the *x* direction. From these results obtained from remote sensing observations, we can see that the spatial distribution of defects is not uniform. Their distribution depends on the sloped direction along which the water flows. The fit of the observations is indicated by continuous orange curves in Fig. 3. Unexpectedly, the *N*(*x*) ∼ log(*x*/*B*)/*x* decay law fits well with the data obtained from Chile and Sudan. For the U.S. landscapes, the fit is excellent.

To understand the complex ecological phenomenon reported above and the role of the law dictating the number density of dislocations in space, mathematical modeling is indispensable. In the following subsections, we investigate the origin of the logarithm decay law through theoretical investigation and numerical simulations of three ecological models.

### Theoretical modeling

To shed light on the observations of the previous section, we consider different standard approaches to explain biomass evolution. The dynamics of ecological systems are often described by either reaction-diffusion models that explicitly incorporate water transport or integrodifferential equations. The latter approach is grounded on nonlocal interactions associated with facilitative and competitive feedback and seed dispersion. Other models based on cellular automata have been first proposed (*14*) and also models based on environmental randomness (*3*, *29*).

We consider the reaction-diffusion (*8*–*11*) and the integrodifferential approaches (*6*, *30*). The later can be seen as a logistic equation with the abovementioned nonlocal interactions, i.e., the spatiotemporal evolution of the normalized biomass *b*(**r**, *t*), reads (*30*).

$$\partial_{t}b=m_{f}(1-b)b-\mu m_{c}b+d\nabla^{2}b$$ (1)

where **r** = (*x*, *y*) and *t* are the spatial coordinates and time, respectively. *m_f_* and *m_c_* account for facilitation and competition plant-to-plant feedbacks. The nonlocal contributions read *m*_*f*,*c*_ = exp [χ_*f*,*c*_ ∫ ‍ ϕ_*f*,*c*_(**r**′)*b*(**r** + **r**′, *t*)*d***r**′], where $\phi_{f,c}(x,y)=\exp[-{\left(x-x_{0f,0c}\right)}^{2}/2l_{fx,cx}^{2}-y^{2}/2l_{fy,cy}^{2}]$ are ellipsoidal coupling kernels with a shift in *x* with respect to the origin of magnitude *x*_0*f*,0*c*_. The facilitative and the competitive ranges are *l*_*fx*,*cx*_ and *l*_*fy*,*cy*_ for the *x* and *y* direction, and the feedback strengths are measured by χ_*f*,*c*_. The Kernels ϕ_*f*,*c*_ introduce an anisotropy and break the reflection symmetry *x* ↔ −*x*. The last term of the right-hand side of Eq. 2 models the seed dispersion with diffusive coefficient *d*. 

In the weak gradient approximation, one can derive from model Eq. 1 a simpler partial differential equation (see Materials and Methods for details) of the form

$$\partial_{t}b=\left(-\eta +\kappa b-\frac{b^{2}}{2}\right)b+p\nabla^{2}b-b(\alpha \partial_{x}+\gamma \partial_{x}^{2}+\partial_{x}^{4})b$$ (2)

where α accounts for the translation parameter of the ellipsoidal kernel. The parameter η measures the decrease–to–growth rate ratio, called the aridity parameter. κ is the facilitation-to-competition strength difference, called the cooperativity parameter. γ is proportional to the difference of the squared competition-to-facilitation lengths and *p* plays the same role as *d*.

In addition to the integrodifferential and the weak-gradient models, we consider the water-biomass model describing the space-time evolution of the biomass (*b*) and water (*w*) density. This model reads (*10*)

$$\begin{array}{c} \partial_{t}b=\left(\frac{\gamma w}{1+\sigma w}-\mu \right)\;b-b^{2}+d\nabla^{2}b\\ \partial_{t}w=p-(1-\rho b)w-w^{2}b+\\ \nabla^{2}(w-\beta b)-\alpha \partial_{x}(w-vb) \end{array}$$ (3)

The slope effect is accounted for in the term α∂*_x_*(*w* − *vb*), where α is the water speed, which flows opposite (in favor) to the *x* direction for α < 0 (α > 0). Because of the water absorption by plants, the biomass reduces the water advective transport mediated by the parameter *v*. The parameters γ and σ model the biomass production increase with water considering a saturable function, *d* models the seed dispersion, and μ accounts for mortality. The parameter *p* measures water input, ρ reduces the transpiration rate linearly with the biomass, and β models how plants affect water absorption by the soil.

**Fig. 4.**
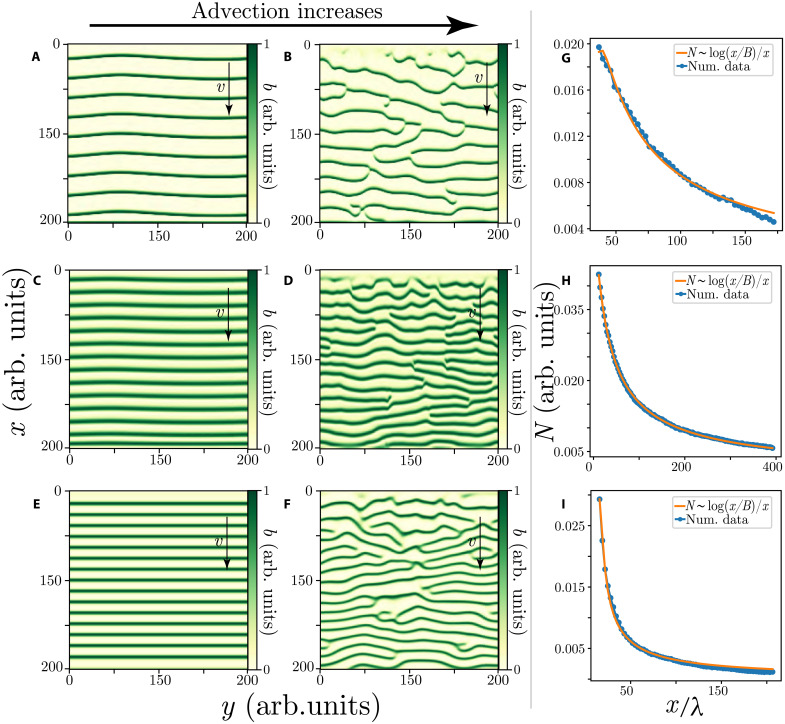
Theoretical modeling of the dislocation decay law: Numerical simulations for three models of migrating banded vegetation patterns with different advection parameters. (**A** and **B**) correspond to the integrodifferential model Eq. 1. Parameters are *l_fx_* = *l_fy_* = 0.5, *l_cx_* = 2.2, *l_cy_* = 0.3, μ = 0.95, χ*_f_* = 2.8, χ*_c_* = 2.0, *d* = 0.01, for (A) *x*_0*f*_ = −0.2 and *x*_0*c*_ = 0.1, for (B) *x*_0*f*_ = −0.4 and *x*_0*c*_ = 0.8. (**C** and **D**) show the weak gradient model Eq. 2, parameters are η = −0.04, κ = 0.3, *p* = 0.05, γ = 1.9, for (C) α = 0.4, for (D) α = 1.0. (**E** and **F**) represent the water-biomass model (3), parameters are γ = 2.0, σ = 1.5, *d* = 0.1, μ = 0.1, *w*_0_ = 0.3, ρ = 0, β = 0, *v* = 4.0, for (E) α = −1.4, for (F) α = −2.0. The right panels correspond to the respective number of dislocations *N*(*x*) as a function of the propagation coordinate (*x*/λ) for each model in the regime of asymptotic uniform stripe patterns. Fit parameters in λ units are (**G**) *x*_0_ = 27.2, *B* = 4.1, and *A* = 0.2 (*R*^2^ = 0.99); (**H**) *x*_0_ = −7, *B* = 3.8, and *A* = 0.5 (*R*^2^ = 1.0); (**I**) *x*_0_ = 14.3, *B* = 0.7, and *A* = 0.06 (*R*^2^ = 0.99).

Numerical simulations of the nonlocal model Eq. 1 with ellipsoidal translated kernels (*l*_*fx*,*cx*_ ≠ *l*_*fy*,*cy*_) display propagative banded patterns for small *x*_0*f*,0*c*_ values as shown in Fig. 4A. These results are obtained using Dirichlet boundary conditions with zero value in the flow direction edges (*b* = 0 for *x* = 0 and *x* = *s*, where *s* is the system size). Periodic boundary conditions are used in the *y* direction. Numerical simulations of all the models presented were conducted with a Runge-Kutta algorithm of fourth order for time integration and a finite difference scheme for space discretization.

As the translation parameter increases, the uniform banded patterns become unstable and the system generates permanent dislocations from the fixed edge *x* = 0, see Fig. 4B. Similarly, a permanent emission of defects can be sustained by environmental stochastic fluctuations (*31*).

**Fig. 5.**
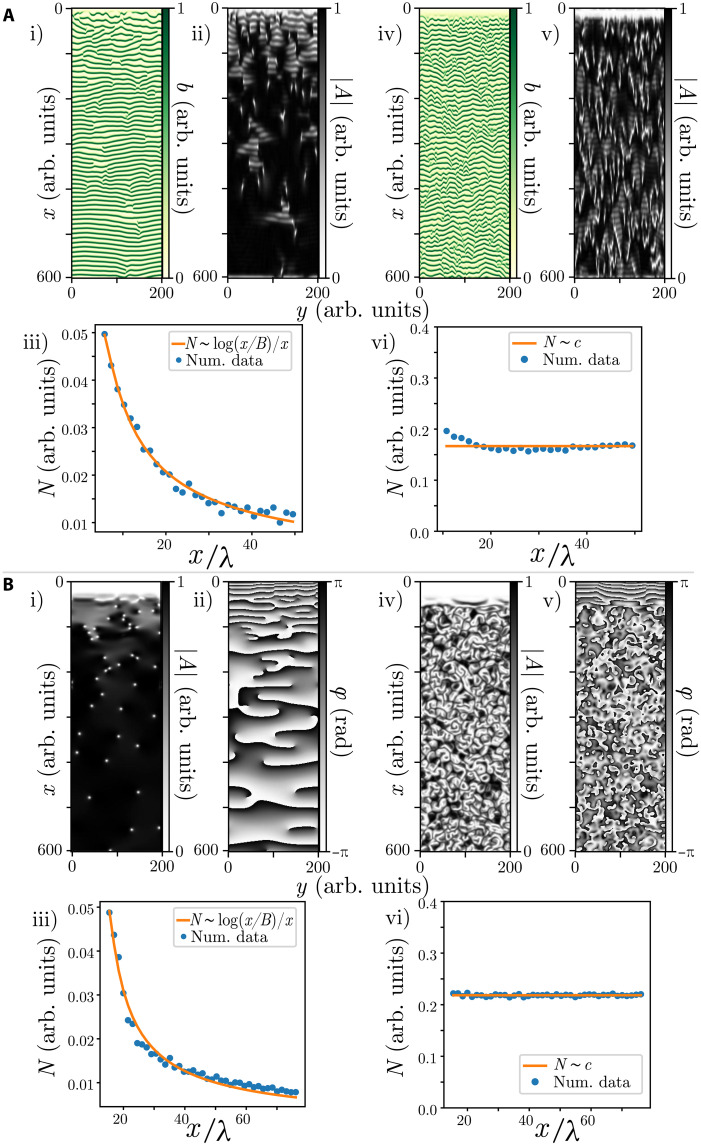
Transition from nonturbulent to turbulent regime. (**A**) Numerical simulations of the weak gradient vegetation model Eq. 2. The real field, the amplitude of the banded pattern, and the defect number distribution. Parameters are η = −0.04, κ = 0.3, *p* = 0.05, γ = 1.9, for (i) and (ii) α = 1.0, for (iv) and (v) α = 2.0. (iii) and (vi) show the number of dislocations *N*(*x*). (**B**) Numerical simulations of the complex Ginzburg-Landau Eq. 9. Parameters μ(*x*) = 1 − *e*^−*x*/10^, ν(*x*) = 10*e*^−*x*/10^, $\tilde{\alpha }=1.0$, for (i) and (ii) β = 0.1, for (iv) and (v) β = 3.0. (iii) and (vi) show the number of dislocations. Fit parameters in λ units are (A, iii) *x*_0_ = 2.7, *B* = 0.9, and *A* = 0.1 (*R*^2^ = 0.99); (B, iii) *x*_0_ = 13, *B* = 0.7, and *A* = 0.1 (*R*^2^ = 0.98). (A, vi) *c* = 0.16; (B, vi) *c* = 0.21

The permanent dislocation dynamics are also obtained from the reduced model Eq. 2 (cf. Fig. 4, C and D) and the reaction-diffusion Eq. 3 (cf. Fig. 4, E and F). All models display a transition from a perfect traveling banded vegetation to a regime where dislocations are permanently emitted, as shown in Fig. 4. This transition occurs for α* < α, the system asymptotically tends to a regular banded pattern as *x* → ∞, cf. Fig. 5A(i), but with dislocations being created in the upstream boundary. The critical value α* is the threshold for the boundary layer instability, and below this value, the number of dislocations is zero. The α* parameter has no analytical expression and depends on the model considered. Hence, this parameter only is determined numerically. When dislocations are only created on the edge, numerical data follows

$$N(x)=\frac{A\log[(x-x_{0})/B]}{\left(x-x_{0}\right)}$$ (4)

where *A*, *B*, and *x*_0_ are the fit parameters. The number of dislocations *N*(*x*) as a function of *x*is plotted in Fig. 5A(iii). This numerical result agrees with field observations using remote sensing image data analysis, as shown in the panels of Fig. 3. Table 1 summarizes the results of fitting law (*4*) to both observational and numerical data.

**Table 1.** Summary of the best fit for the decaying spatial distribution of dislocations for mathematical models and remote sensing image analysis. *A*, *B*, and *x*_0_ are the fit parameters of Eq. 4, and *R*^2^ is the coefficient of determination for the respective fits.

Mathematical models	*A*/λ	*B*/λ	*x*_0_/λ	*R* ^2^
Eq. 1	0.2	4.1	27.2	0.99
Eq. 2	0.5	3.8	−7.0	1.0
Eq. 3	0.06	0.7	14.3	0.99
Remote sensing image analysis				
Chile	2.7	1.2	−2.9	0.92
Sudan	10.6	1.5	−2	0.91
United States	5	1.3	−2.4	0.96

To understand analytically the origin of the logarithmic law, we perform a normal form analysis, which leads to the derivation of the well-known Ginzburg-Landau Eq. 9 (see Materials and Methods). Dislocations correspond to topological singularities in the phase of the Ginzburg-Landau equation (*32*–*34*). This reduction shows that in defect interaction, when the nonlinear phase correction β is small, the length *l* between the defects decays according to the law *l*^2^ = *t*/log(*t*) (*35*–*37*). Then if the system is advected with speed α, one can interchange the role of time for space using the relation *t* = *x*/α. Hence, this characteristic length changes with distance as *l*^2^ = *x*/αlog(*x*/α). Likewise, the average number of defects in a given area Π is *N*(*x*) = Π/*l*^2^ = Παlog(*x*/α)/*x*. Again, the normal form analysis confirms the logarithmic law.

When, however, the advection parameter increases, i.e., large α, we identify a second transition where a permanent creation of dislocations occurs not only from the edge but also in the bulk, as shown in Fig. 5A(iv). This regime is well-known in nonlinear systems in general, and it is referred to as defects turbulence (*32*, *38*). In this regime, the averaged dislocation number is constant *N*(*x*) = *c* as a result of the continuous creation of defects in the bulk, see Fig. 5A(vi). This figure is obtained from numerical simulations of Eq. 2. The transition from nonturbulent to turbulent regime is also obtained from the Ginzburg-Landau Eq. 9, as shown in Fig. 5B.

The numerical analysis of ecological models indicates that by only measuring the number of dislocations in the pattern, one can infer if the semi-arid and arid ecosystems operate in the turbulent regime where *N*(*x*) = *c* or in the nonturbulent regime where *N*(*x*) obeys a logarithmic decay law. This law obtained from numerical simulations of the three models considered here is in good agreement with observations using remote sensing image analysis, as shown in the panels of Fig. 3. Therefore, the measure of the number of dislocations in the vegetation patterns and their spatial distribution can be used as a noninvasive tool for diagnosing the degree of complexity of arid landscapes and for identifying unexpected dynamical phenomena in ecological systems.

## DISCUSSION

**Fig. 6.**
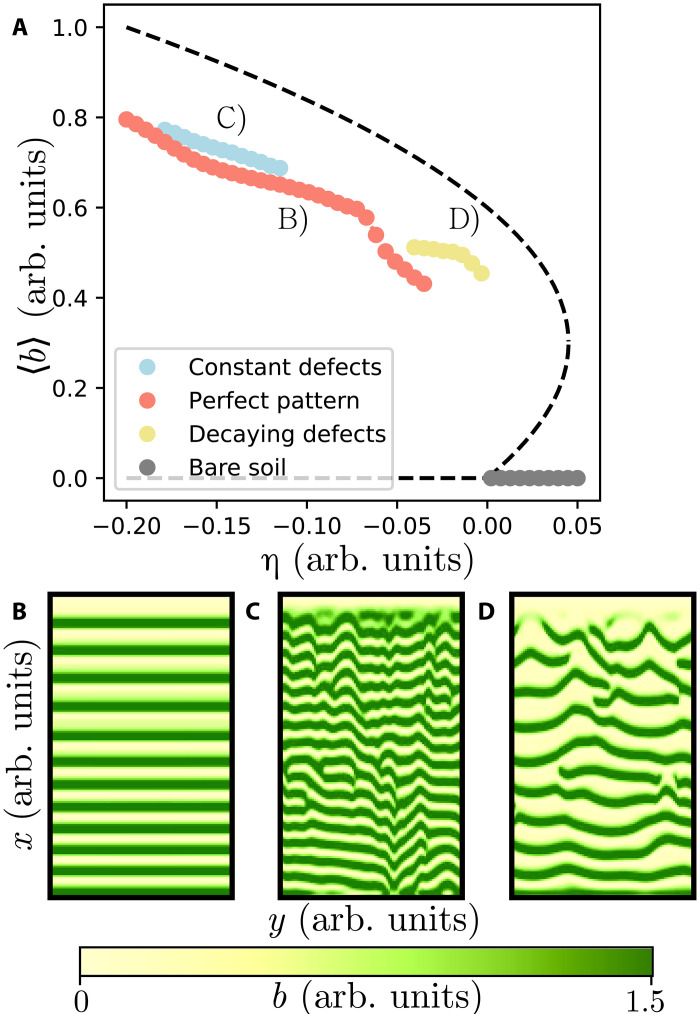
Diagram of migrating banded vegetation pattern biomass as a function of the aridity parameter η. For Eq. 2, parameters κ = 0.3, *p* = 0.05, γ = 2, and α = 1. (**A**) illustrates the mean biomass 〈*b*〉 at the steady states of the model when changing the aridity. (**B**) corresponds to the branch of perfect patterns *N*(*x*) = 0. (**C**) shows the turbulent-like behavior where *N*(*x*) = *c*. (**D**) represents the branch of asymptotic patterns, where *N*(*x*) ∼ log(*x*/*B*)/*x*.

The transition between different regimes is investigated in terms of the speed of the water flows. We have shown that for a large speed, the ecosystem presents a turbulent behavior where the number of topological defects is constant. For a small value of the water flow speed, the number of defects decreases according to the logarithmic law. In what follows, we discuss the effect of the aridity level on dislocation formation. For this purpose, we fix the speed of the water flow and vary the aridity level. Figure 6 summarizes the different ecosystem operating regimes. For small aridity parameters, the system develops migrating banded pattern devoid of defects (cf. red curve). For a moderate level of aridity, the system exhibits a transition toward a turbulent regime where the number of dislocations is constant (see blue curve). However, for extreme aridity conditions, the system reaches a regime where the system undergoes self-organized dislocations with a logarithmic decay law (see yellow curve). Further increasing the aridity, the banded patterns exhibit a transition toward a state totally devoid of vegetation. Thus, for a given landscape with a homogeneous slope, the presence of a decaying number of dislocations can be an ecological indicator of imminent transition toward a bare state. This complements what is known about the catastrophic shift of ecosystems in flat topography, where different types of stationary patterns exist and where multistability of patterns with different wavelengths can be observed. The existence of many pattern branches permits the ecosystem to adapt to environmental changes, which allows a patterned ecosystem to survive past the tipping point compared to a homogeneous ecosystem (*22*). However, once a gentle slope is introduced into the system, the advective effects of water flow must be taken into account for the stability analysis of patterns and other solutions of the system (*39*). This changes the stable pattern branches compared to a flat territory case. In addition, complex and turbulent-like dynamics can emerge as a consequence of the slope. These complex dynamical regimes have their own relative stability compared to the different perfect migrating pattern regimes and homogeneous states. Multistability of the complex dynamical regimes and the perfect patterns can occur, as observed in Fig. 6, suggesting that in adaptation to change, ecosystems could transit to these complex regimes if in the presence of a slopped territory. Numerically, only the most stable branch of perfect patterns is accessible. Different stable branches of perfect patterns could be obtained from Eq. 9 analytically at the onset of pattern formation.

To summarize, we have investigated different transitions of migrating vegetation banded patterns: from zero defects, to constant, and to a decaying number of dislocations. We have shown analytically that the number of dislocations in space *N*(*x*) obeys a *N*(*x*) ∼ log(*x*/*B*)/*x* law. This formula is in good agreement with numerical simulations of the three ecological models and with remote sensing image data taken from three arid ecosystems of different continents. Furthermore, the dislocation law allows us to determine whether the self-organized response to the water scarcity of arid and semi-arid ecosystems favors uniform bands or ecological spatio-temporal complexity.

A usual approach to characterize the response of plants to changes in their environment is through studies of the plants themselves (local analysis). Characterizing dislocation distributions of migrating banded vegetation patterns (macroscopic analysis) opens a noninvasive diagnostic tool for determining the degree of aridity mediated by desertification and global warming processes. Likewise, a full characterization of the bifurcation diagram for models including reflection symmetry rupture and dislocations self-organization becomes relevant in designing conservation guidelines, preventing the further degradation of migrating patterned vegetation cover.

Last, the spatial distribution of defects is a consequence of their creation induced by the boundary condition and their annihilation through mutual interaction. The boundary induces inhomogeneities in the system. The inhomogeneous Ginzburg-Landau equation constitutes an ideal framework for investigating the dynamics of defects in banded vegetation patterns. It provides a unified and simple description containing the dynamics discussed. Thus, the analytical results can be easily extended to describe similar laws in other complex nonlinear spatially extended systems present in nature.

## MATERIALS AND METHODS

### Detailed derivation of the weak gradient model with advection

We look for an approximation to Eq. 1 of the main text, in the form of a partial differential Eq. 2. To account for anisotropy, we consider that the interaction ranges associated with facilitation and the competition *l*_*cx*,*fx*_ and *l*_*cy*,*fy*_ are different. We seek corrections to the steady states close to μ = 1 and *b* = 0 that depend on time and space through the slow variables *T* = ε*t*, *X* = ε^1/8^*x*, and *Y* = ε^1/8^*y*. We expand the parameters μ, χ_*f*,*c*_, *l*_*fx*,*fy*_, *x*_0*c*,0*f*_, *d*, and the biomass *b* in terms of a small parameter ε (ε ≪ 1) that measures the distance from μ = 1 as follows

$$\begin{array}{c} \mu =1+\epsilon \eta +\ldots ,\\ \chi_{f}=1+\chi_{c}+\epsilon^{1/2}\kappa +\ldots ,\\ \chi_{c}=\frac{l_{fx}^{2}}{l_{cx}^{2}-l_{fx}^{2}}+\epsilon^{1/4}\chi_{1}+\ldots ,\\ x_{0f}=\epsilon^{3/8}\alpha_{f}+\ldots ,\;x_{0c}=\epsilon^{3/8}\alpha_{c}+\ldots ,\\ l_{fy}^{2}=\epsilon^{1/4}\sigma_{fy}^{2}+\ldots ,\;l_{cy}^{2}=\epsilon^{1/4}\sigma_{cy}^{2}+\ldots ,\\ d=\epsilon^{3/4}p+\ldots ,\;b(t,x,y)=\epsilon b(T,X,Y)+\ldots \end{array}$$

Introducing these scalings and the above expansions in Eq. 1, we then obtain a sequence of linear problems for unknown functions. We analyze each problem and apply the solvability condition at each order. These conditions are automatically fulfilled at the orders ε^1/2^ and ε. By applying the solvability condition at the higher order inhomogeneous problem (ε^3/2^), we obtain the following partial differential equation for the biomass

$$\begin{array}{c} \partial_{T}b=(-\eta +\kappa b-b^{2}/2)b+p\nabla^{2}b\\ -b(\alpha \partial_{X}+\gamma_{x}\;\partial_{X}^{2}-\gamma_{y}\;\partial_{Y}^{2}+\text{Λ}\partial_{X}^{4})b \end{array}$$ (5)

where the coefficients are $\chi_{0}=l_{fx}^{2}/(l_{cx}^{2}-l_{fx}^{2})$, α = α*_c_*χ_0_ − α*_f_*(1 + χ_0_), $\gamma_{x}=\chi_{1}(l_{cx}^{2}-l_{fx}^{2})$, $\gamma_{y}=\sigma_{fy}^{2}(1+\chi_{0})-\sigma_{cy}^{2}\chi_{0}$, and $\text{Λ}=3l_{fx}^{2}l_{cx}^{2}$.

Model Eq. 5 has different homogeneous steady states which are *b* = 0 and *b*_0_ = κ ± (κ^2^ −2η)^1/2^. Note that the upper branch of *b*_0_ is stable when *l_cx_* > *l_fx_* and γ*_y_* > 0. Otherwise, we need to consider higher ε orders in the equation. The condition γ*_y_* = 0 will be used throughout the work, as it does not change the qualitative behavior of the system.

Introducing the scaling $X=1/{\text{Λ}}^{\frac{1}{4}}X\;\mathrm{and}\;\;Y=1/{\text{Λ}}^{1/4}Y$and redefining α → α/Λ^1/4^, γ*_x_* → γ/Λ^1/2^, and *p* → *p*/Λ^1/2^, we get Eq. 2.

### Detailed derivation of the Ginzburg-Landau equation

#### Derivation of amplitude equation in the bulk

The amplitude equation obtained using a normal form analysis constitutes an adequate tool for understanding pattern formation. For the boundary conditions considered, the system creates a thin boundary layer next to the upstream edge of the system. The effect of this boundary layer can be neglected when focusing on regions far from the edges. Let us consider first the linear problem for a perturbation of the homogeneous stable state *u* ≪ 1 as *b* = *b*_0_ + *u*, where $b_{0}=\kappa +\sqrt{\kappa^{2}-2\eta }$ is the homogeneous cover. Introducing this ansatz in Eq. 2 of the main text for the field *b* yields the linear problem

$$(\partial_{t}-L)u=0$$

where the linear operator is defined as $L\equiv -\eta +2\kappa b_{0}-3b_{0}^{2}/2+p\nabla^{2}-b_{0}(\alpha \partial_{x}+\gamma \partial_{x}^{2}+\partial_{x}^{4})$. Linear stability analysis for finite wavenumber *k* perturbations leads to the growth rate of modes λ(*k*)

λ(k)=Reλ(k)+iΩ(k)=−η+2κb0−3b022−pk2−b0(iαk−γk2+k4)

The conditions ∂*_k_*Reλ∣*_k_c__* = 0 and Reλ(*k_c_*) = 0 determine the critical wavenumber $k_{c}^{2}=(b_{c}\gamma -p)/2b_{c}$ and the critical aridity parameter, which satisfies $-\eta_{c}+2\kappa b_{c}-3b_{c}^{2}/2+b_{c}k_{c}^{4}=0$, where $b_{c}=\kappa +\sqrt{\kappa^{2}-2\eta_{c}}$. To obtain the amplitude equation for the critical mode, let us move slightly from the instability condition, using as the bifurcation parameter η, as η = η*_c_* + ε. Introducing the following expansion

$$b=b_{0}+\epsilon^{1/2}Ae^{ik_{c}x+i{\text{Ω}}_{c}t}+\epsilon A^{\left[2\right]}+\epsilon^{3/2}A^{\left[3\right]}+.\cdots +c.c.$$

where *A* ≡ *A*(*X*, *Y*, *T*) is the slowly varying envelope, with the scalings *X* = ε^1/2^*x*, *Y* = ε^1/2^*y*, *T* = ε*t*, and the parameter Ω*_c_* ≡ −*b_c_*α*k_c_*. *A*^[*n*]^ accounts for the terms of order *n* in the amplitude *A*. At order (ε^1/2^), we obtain

$$(\partial_{t}-L_{c})Ae^{ik_{c}x+i{\text{Ω}}_{c}t+\lambda (k_{c})t}=0$$

where

$$L_{c}=-\eta_{c}+2\kappa b_{c}-\frac{3b_{c}^{2}}{2}+p\nabla^{2}-b_{c}(\alpha \partial_{x}+\gamma \partial_{x}^{2}+\partial_{x}^{4})$$

At this order, the solvability condition is automatically satisfied. For the sake of simplicity, let us define $\hat{d}\equiv \alpha \partial_{x}+\gamma \partial_{x}^{2}+\partial_{x}^{4}$, and *d*(*k*) ≡ *i*α*k* − γ*k*^2^ + *k*^4^. Then, performing expansions, up to order ε, limiting to the case of small group velocities *v_g_* = ∂*_k_*Ω∣*_k_c__* ∼ *O*(ε^1/2^), we get

$$\begin{array}{c} \left(\partial_{t}-L_{c})A^{\left[2\right]}=\left(\kappa -\frac{3}{2}b_{c}\right)\;(A^{2}e^{2{ik}_{c}x+2i{\text{Ω}}_{c}t}+{|A|}^{2}+c.c.\right)\\ -({Ae}^{ik_{c}x+i{\text{Ω}}_{c}t}+c.c.)\;[Ad(k_{c})e^{ik_{c}x+i{\text{Ω}}_{c}t}+c.c.] \end{array}$$ (6)

To solve the linear problem, the following inner product is introduced

$$\langle f|g\rangle =\int_{X}^{X+\frac{2\pi }{k_{c}}}dx\int_{T}^{T+\frac{2\pi }{{\text{Ω}}_{c}}}dtf^{*}g$$

valid over the periodic functions in space and time of period 2π/*k_c_* and 2π/Ω*_c_*. The kernel of the operator (∂*_t_* − *L_c_*)^†^, defined as the solution of (*∂_t_* − *L_c_*)^†^ψ = 0, corresponds to ψ = *e*^±*i*(*k_c_x*+Ω*_c_t*)^. Then, applying the solvability condition, we find

$$A^{\left[2\right]}=a_{2}A^{2}e^{2ik_{c}x}+b_{2}{|A|}^{2}+{\bar{a}}_{2}{\bar{A}}^{2}e^{-2ik_{c}x}$$

where

$$a_{2}=\frac{\kappa -3/2b_{c}-d(k_{c})}{2i{\text{Ω}}_{c}-\lambda (2k_{c})}$$

$$b_{2}=\frac{2\kappa -3b_{c}-d(k_{c})-d(-k_{c})}{-\lambda (0)}$$

Last, at order ε^3/2^, the solvability condition yields

$$\begin{array}{c} \partial_{T}A=\mu A+(a+i\beta ){|A|}^{2}A\\ +D_{x}\;\partial_{XX}A+D_{y}\;\partial_{YY}A-\alpha b_{c}\;\partial_{X}A \end{array}$$ (7)

with ${\mu =(-1+2\kappa \partial_{\eta }b_{0}-3/2\partial_{\eta }b_{0}^{2}+(\partial_{\eta }b_{0})k_{c}^{4}+b_{c}\partial_{\eta }k_{c}^{4})|}_{\eta_{c}}$, (*a* + *i*β) = (2η*_c_* − 3*b_c_*)(*b*_2_ + *a*_2_) − 3/2 − *a*_2_*d*(2*k_c_*) − *a*_2_*d*(−*k_c_*) − *b*_2_*d*(*k_c_*), $D_{x}=4b_{c}k_{c}^{2}$, and *D_y_* = *p*. This equation is the well-known Ginzburg-Landau equation with advection.

#### Boundary layer effect

To figure out the emission of dislocations from the boundary of the system in the regime of decaying number of dislocations, we need to consider the boundary layer effect arising from the Dirichlet boundary conditions. We use the method suggested in (*40*). For this purpose, we suppose that sufficiently near to the upstream edge, one can write

$$b=b_{0}+\epsilon M(X)$$

where *M*(*X*) is a function that helps to connect the population state *b* = *b*_0_ with the nonpopulation state *b* = 0 at the boundary and satisfies *M*(*X*) → 0 when *X* → ∞. The analytical solution close to the boundary is not known. Qualitatively, *b*(*X*) is a Monod function. On the basis of this nonuniform *b*, a modified amplitude equation is derived. Making straightforward calculations, one finds a similar amplitude equation compared to Eq. 7 but with inhomogeneous linear terms

$$\begin{array}{c} \partial_{T}A=[\mu +\mu_{1}\;(X)+i\nu (X)]\;A+(a+i\beta ){|A|}^{2}\;A\\ +D_{x}\;\partial_{XX}A+D_{y}\;\partial_{YY}A-\alpha b_{c}\;\partial_{X}A \end{array}$$ (8)

where the parameters depend on *M*(*X*) as

$$\mu_{1}(X)=(2\kappa -3b_{c}+\gamma k_{c}^{2}-k_{c}^{4})M(X)$$

$$\nu (X)=\alpha k_{c}M(X)$$

Both terms are proportional to the slow inhomogeneity *M*(*X*), so they asymptotically go to 0 as *X* → ∞. Hence, one recovers the homogeneous Ginzburg-Landau Eq. 7. With the change of parameters and variables as $X=\sqrt{D_{x}}X$, $Y=\sqrt{D_{y}}Y$, $A=1/\sqrt{-a}A$, β = β/*a*, and $\tilde{\alpha }=b_{c}\alpha /\sqrt{D_{x}}$, we get Eq. 9.

### Amplitude equation description

The amplitude of migrating patterns *A* satisfies the complex Ginzburg-Landau equation with advection

$$\partial_{t}A=[\mu (x)+i\nu (x)]A-(1+i\beta ){|A|}^{2}A+\nabla^{2}A-\tilde{\alpha }\partial_{x}A$$ (9)

The bifurcation parameter μ = μ(*x*) and detuning ν = ν(*x*) are inhomogeneous as a consequence of the boundary layer. For the sake of simplicity, we choose *M*(*x*) ∼ *e*^−*x*^, where *x* = 0 accounts for the position of the upstream edge, cf. Fig. 5B.

Note that similarly to ecological models described above, the amplitude Eq. 9 supports a permanent emission of dislocations from the upstream edge caused by the inhomogeneous character of μ and ν. The modulus, the phase field, and the dislocation distribution for the nonturbulent and turbulent regimes are shown in Fig. 5B. Both defect number laws *N*(*x*) are consistent with vegetation models’ predictions.

### Defect counting in numerical simulations

Reconstructing the analytical signal of the migrating vegetation patterns presented, dislocations are recognized as zeros of the amplitude field. This is achieved by the binarization process of the amplitude field and a particle detection algorithm, both available in the Fiji software (*28*). This software gives the position of all the particles (closed regions of zero amplitude) from which *N*(*x*) is constructed. The permanent emission of dislocations enables us to compute *N*(*x*). Using several snapshots of the time evolution for different initial conditions, the mean value of *N*(*x*) is obtained. Note that dislocations start to be counted after the boundary layer region where they are created.

### Remote sensing data analysis

#### Image treatment

Satellite images are processed with Fiji (*28*). Grayscale images are treated with a one-pixel width Gaussian blur to reduce inhomogeneities. Then, the subtract background algorithm with a rolling ball of a radius of 10 pixels is applied. Last, the image is binarized and a skeleton is constructed. Note that all the procedure is easily implemented with prebuilt Fiji functions.

#### SRTM data analysis

SRTM data are obtained from the public database (*25*). The netCDF4 files are analyzed in Python with the netCDF4 module. The height maps are given with one–arc sec resolution in both the azimuthal and polar angles; thus, localizing the bounding coordinates of the regions of interest (with Google Earth software) allows obtaining the topography of the desired regions. Then, the gradient of the height map is calculated numerically to obtain the steepest direction at each point. Last, this direction is averaged over the region of interest, and the mean orientation 〈θ〉 is obtained. This angle is used to rotate the previously obtained skeletons, aligning the *x* direction (the steepest descent) with the horizontal or vertical axis. This allows for an efficient way to count dislocations.

#### Defect counting

One needs to consider that the region of interest to analyze is not rectangular as the ones obtained from numerical simulations. Thus, a density of defects is computed to consider irregularities in the region of interest boundaries. For example, consider that *x* and *y* are aligned with the *i* and *j* indices of the matrix representing the image, and then, for each *j*, we swept the *i* index in search of defects to construct a density *n*(*x*, *y*). This is a binary function of (*x*, *y*), with zero value if no dislocation is found and one if there is a dislocation. Their distance is measured from the boundary of the column analyzed, which is given by the mask of the region of interest. Note that for a single column *j*, there can exist several boundaries due to complex topography, if this is the case, subsequent intervals are treated as new columns. Last, we coarse grain the density in tiles of one wavelength sides and average over the *y* direction, obtaining *N*(*x*).
